# Research progress on the key cuproptosis regulator FDX1 and its relevance to triple-negative breast cancer

**DOI:** 10.3389/fmolb.2026.1985506

**Published:** 2026-10-20

**Authors:** Fan Yuping, Zeng Hongmiao, Yuan Songlin, Pu Chenchen, Zhu Kepeng

**Affiliations:** 1 Department of Breast and Thyroid Surgery, the Affiliated Nanchong Central Hospital of North Sichuan Medical College, Nanchong, Sichuan, China; 2 The People’s Hospital of Tianfu New District, Chengdu, Sichuan, China

**Keywords:** cuproptosis, FDX1, mitochondrial metabolism, triple-negative breast cancer (TNBC), tumor immune microenvironment

## Abstract

Cuproptosis, a recently identified form of programmed cell death driven by copper ion overload, occurs in a manner highly dependent on mitochondrial respiration and is orchestrated by the key reductase FDX1 (Ferredoxin 1). Triple-negative breast cancer (TNBC), the most aggressive subtype of breast cancer, lacks expression of the estrogen receptor, progesterone receptor, and human epidermal growth factor receptor 2 (HER2), rendering patients ineligible for endocrine therapy and anti-HER2 targeted treatments. Consequently, there is an urgent need to develop novel therapeutic strategies for this malignancy. The discovery of cuproptosis offers a fresh perspective for TNBC research: the prevalent metabolic reprogramming and hyperactive mitochondrial function characteristic of TNBC cells constitute an intrinsic vulnerability that may predispose them to cuproptosis induction. Emerging evidence indicates that low FDX1 expression may correlate with poor prognosis in TNBC, However, current evidence remains largely confined to mechanistic studies, bioinformatics analyses, and limited models; whether FDX1 expression correlates with prognosis in TNBC patients still awaits clinical translational validation.This review aims to systematically summarize the latest advances in research on the key cuproptosis regulator FDX1 and its relevance to TNBC, with the goal of providing directions for targeted therapeutic investigations in TNBC.

## 1 Introduction

Triple-negative breast cancer (TNBC) is defined as a breast cancer subtype characterized by the absence of estrogen receptor (ER), progesterone receptor (PR), and human epidermal growth factor receptor 2 (HER2), accounting for approximately 15%–20% of all breast cancers (Garrido-Castro et al., 2019).This “triple-negative” feature renders TNBC unresponsive to endocrine therapy and anti-HER2 targeted treatments. At the molecular level, TNBC exhibits substantial heterogeneity; clinically, it is distinguished by markedly more aggressive behavior, higher risk of early relapse, and increased distant metastasis (particularly to the lung and brain) compared with other subtypes (Foulkes et al., 2010). Although TNBC demonstrates initial sensitivity to chemotherapy, patients are highly prone to developing drug resistance, and treatment options become extremely limited upon recurrence. In recent years, immune checkpoint inhibitors (ICIs) have achieved breakthroughs in a subset of PD-L1-positive patients; however, challenges of resistance and relapse persist, and the overall survival of TNBC patients remains significantly inferior to that of other subtypes (Li et al., 2025). The pronounced heterogeneity and distinctive metabolic reprogramming of TNBC confer substantial resistance to conventional therapeutic strategies primarily aimed at inducing apoptosis. Consequently, the exploration of novel mechanisms of non-apoptotic programmed cell death (PCD) has emerged as a critical scientific imperative for overcoming the therapeutic bottleneck in TNBC.

In recent years, the discovery of novel forms of programmed cell death (PCD), including ferroptosis, pyroptosis, and necroptosis, has provided entirely new perspectives for cancer therapy. In 2022, (Tsvetkov et al., 2022) ormally defined cuproptosis for the first time, revealing a unique mechanism by which copper ions induce cell death through targeting mitochondrial metabolism. Unlike apoptosis, necroptosis, and other forms of cell death, cuproptosis is driven by the direct binding of excess copper ions to lipoylated proteins in the mitochondrial tricarboxylic acid (TCA) cycle, such as dihydrolipoyl S-acetyltransferase (DLAT) and dihydrolipoyl S-succinyltransferase (DLST). This binding triggers abnormal oligomerization and functional loss of these proteins, leading to proteotoxic stress, and ultimately resulting in mitochondrial collapse and cell death. Within this core regulatory network, FDX1 has been identified as the direct upstream regulator of cuproptosis (Lu et al., 2025; Wu et al., 2025a; Liu and Miao, 2023), By reducing Cu^2+^ to the more toxic Cu^+^, driving protein lipoylation modification, and disrupting iron-sulfur cluster homeostasis, FDX1 serves as the “molecular switch” that initiates cuproptosis. The expression level and functional status of FDX1 directly determine the sensitivity of tumor cells to cuproptosis, establishing it as a focal molecule in this field of research.

Pan-cancer analyses have revealed that FDX1 exerts tumor-suppressive effects in various malignancies, including clear cell renal cell carcinoma and hepatocellular carcinoma, and its expression is closely associated with immune cell infiltration and immunotherapy response, demonstrating dual functions in metabolic regulation and immune modulation (Liu and Miao, 2023). However, in the specific context of TNBC, research on FDX1 presents a uniquely complex picture: although FDX1 mRNA levels are significantly lower in TNBC than in other subtypes and correlate with poor prognosis (Jing et al., 2025),imple overexpression of FDX1 fails to effectively induce cuproptosis in TNBC cells, suggesting the existence of sophisticated post-transcriptional or post-translational regulatory mechanisms (Liu and Miao, 2023). One study reported (Drobnie et al., 2025)that over 90% of TNBC cases harbor TP53 mutations, and that p53 loss relieves the transcriptional repression of mitochondrial metabolism, copper homeostasis, and FDX1-related lipoylation pathways, thereby markedly enhancing mitochondrial metabolism. Moreover, TNBC is characterized by high AKT1 activation, and AKT1 can directly phosphorylate FDX1 to abrogate its function and block cuproptosis, constituting a TNBC-specific mechanism of cuproptosis resistance (Sun et al., 2025). Although FDX1 is not a TNBC-exclusive marker *per se*, the combined context of the TNBC genomic background, copper metabolism, AKT signaling, and the immune microenvironment renders its effects on survival, invasion, and immune evasion in TNBC far more prominent than in most solid tumors.In light of this, the present review will focus on dissecting the biological characteristics of FDX1, its expression regulatory mechanisms, and its specific expression patterns and clinical translational value in TNBC, with the aim of providing theoretical support and practical direction for the development of cuproptosis induction-based precision therapeutic strategies for TNBC.

## 2 Biological characteristics of FDX1

### 2.1 Molecular structural features and physiological functions of FDX1

FDX1 is a small iron-sulfur (Fe-S) cluster protein localized to the mitochondria. The human FDX1 gene is mapped to the q22 region of chromosome 11 (Sheftel et al., 2010), comprises four exons (Cunningham et al., 2022), and encodes a protein of 184 amino acids with a molecular weight of 19.4 kDa (He et al., 2026). FDX1 participates in a variety of biological functions, including protein lipoylation, iron-sulfur cluster biogenesis, steroid and bile acid synthesis, formation of heme α/α3, metabolism of vitamins A and D, and copper ion reduction (Sheftel et al., 2010). Its core functions are primarily manifested in three aspects: steroid biosynthesis, protein lipoylation, and cuproptosis. During steroidogenesis, FDX1 receives electrons from NADPH *via* ferredoxin reductase (FDXR)—an NADPH-dependent flavoprotein—and transfers them to mitochondrial cytochrome P450 enzymes (CYPs), thereby initiating steroid biosynthesis. In terms of protein lipoylation, FDX1 not only supports mitochondrial metabolism but also generates the substrates required for cuproptosis (Dreishpoon et al., 2023). Under conditions of copper ion overload, FDX1 reduces Cu^2+^ to Cu^+^, promotes the aggregation of lipoylated proteins, and inhibits iron-sulfur cluster biogenesis, ultimately inducing cuproptosis. Therefore, FDX1 serves as a critical mediator of cuproptosis. In summary, FDX1, functioning as an electron transfer protein and reductase, is extensively involved in multiple metabolic pathways and plays a pivotal role in the process of cuproptosis.

### 2.2 Tissue expression characteristics of FDX1

FDX1 is widely expressed in normal human tissues, with the highest expression observed in adrenal tissue, followed by kidney and testis (Li X. et al., 2022), which is highly consistent with the classical function of FDX1 in steroid synthesis. In contrast, FDX1 protein levels are extremely low or even undetectable in most other normal tissues. In tumor tissues, FDX1 expression exhibits dual characteristics of tissue specificity and cancer type specificity: it is downregulated in triple-negative breast cancer, lung adenocarcinoma, hepatocellular carcinoma, renal cell carcinoma, and colorectal cancer, where its low expression is associated with poor prognosis, metastasis, and resistance to cuproptosis (Liu and Miao, 2023; Sun et al., 2025; Li X. et al., 2022; Huang et al., 2022); onversely, it is upregulated in glioblastoma and ovarian cancer, where its high expression positively correlates with malignancy and invasive capacity (Huang et al., 2023; Gong et al., 2026).

### 2.3 Regulatory mechanisms of FDX1 expression

The transcription of FDX1 is precisely regulated by multiple transcription factors, which can exert either activating or inhibitory effects depending on the cancer type. Transcriptional repressors: YY1 (Meliala et al., 2020) suppresses FDX1 transcription through the MAPK-ERK1/2-YY1 signaling axis, The underlying mechanism is that ERK pathway activation promotes YY1 nuclear translocation, and YY1 directly acts on the FDX1 promoter to suppress FDX1 transcription.functioning as a transcriptional inhibitor in ovarian cancer and hepatocellular carcinoma; NRF2 (Wu C. et al., 2025) Under oxidative stress, NRF2 translocates into the nucleus and directly suppresses FDX1 transcription, thereby inhibiting cuproptosis.as a core transcription factor in oxidative stress responses, inhibits FDX1 expression in cervical cancer cells; FOXO3 (Wang et al., 2023) Activation of the upstream PI3K/AKT pathway phosphorylates FOXO3, phosphorylated FOXO3 translocates into the nucleus represses FDX1 transcription in clear cell renal cell carcinoma, cuproptosis is blocked. Transcriptional activators: In a mouse Leydig cell model (Roumaud et al., 2017), steroidogenic factor SF-1 was shown to synergize with c-JUN to activate the FDX1 gene promoter, thereby driving the occurrence of cuproptosis; In glioblastoma, inflammatory signaling factors induce NF-κB nuclear translocation, where NF-κB directly binds to the FDX1 promoter and, as a positive transcriptional regulator, upregulates FDX1 expression (Wu et al., 2025c); In summary, YY1, NRF2, and FOXO3 function as transcriptional repressors of FDX1, whereas SF-1 and NF-κB act as transcriptional activators, collectively constituting a complex regulatory network governing FDX1 transcription. The regulation of FDX1 transcription by YY1, NRF2, FOXO3, SF-1, and NF-κB is highly cell type-specific, post-translational modification-dependent, and microenvironment-dependent. There is no unified, universally applicable activation–inhibition mode. Instead, the ultimate expression level of FDX1 reflects the combined outcome of competition and synergy among multiple transcription factors, rather than the standalone influence of any one factor.

Epigenetic modifications: FDX1 expression is regulated by multiple epigenetic mechanisms, primarily including DNA methylation, histone modifications, and lactylation. Regarding DNA methylation, FDX1 gene expression is negatively correlated with methylation levels in its promoter region across various cancers, and hypermethylation of the promoter can lead to transcriptional silencing of FDX1. In terms of histone modifications, studies have demonstrated (Ma et al., 2025) that LncRNA PVT1 can activate FDX1 transcription by increasing the acetylation level of histone H3 at lysine 27 (H3K27ac). Additionally, lactylation modification is also involved in the regulation of FDX1 expression: upon lactylation at the K229 site, methyltransferase-like protein 16 (METTL16) exhibits enhanced methyltransferase activity, which subsequently upregulates FDX1 expression through m^6^A modification of FDX1 mRNA, ultimately promoting cuproptosis in gastric cancer cells (Sun et al., 2023). Evidence linking METTL16 lactylation to FDX1 regulation through m^6^A modification is derived exclusively from gastric cancer. Whether this axis operates in triple-negative breast cancer remains to be validated, representing a current gap in the field.

Non-coding RNA regulation: FDX1 expression is also subject to multi-level regulation by non-coding RNAs (ncRNAs). ncRNAs primarily comprise three categories: microRNAs (miRNAs), long non-coding RNAs (lncRNAs), and circular RNAs (circRNAs) (Anastasiadou et al., 2018). Studies have found that miRNAs mainly inhibit FDX1 expression by directly binding to the 3′-UTR of FDX1 mRNA in hepatocellular carcinoma, clear cell renal cell carcinoma, and non-small cell lung cancer (Xu W. et al., 2025; Sun et al., 2026). The regulatory mechanisms of lncRNAs on FDX1 are more complex, encompassing both direct activation of transcription by acting on promoter regions and indirect regulation through the competing endogenous RNA (ceRNA) mechanism. For instance, PVT1 (Ma et al., 2025) directly binds to the FDX1 promoter and recruits SF-1, thereby promoting FDX1 transcription through epigenetic activation in colorectal cancer; lncRNA 02362 (Quan et al., 2023) indirectly upregulates FDX1 expression in hepatocellular carcinoma by reducing miR-18-5p levels; MALAT1 (Ding et al., 2023) increases FDX1 expression in gastric adenocarcinoma by suppressing miR-328-3p. Circular RNAs also participate in the regulation of FDX1 expression; a specific circKIAA1797 (8-oxoguanine-modified form) (Xu H. et al., 2025) can bind to FDX1 mRNA and reduce its stability, thereby inhibiting cuproptosis and promoting lung cancer progression.

In addition to transcriptional regulation, ubiquitin–proteasome-mediated degradation of FDX1 protein constitutes an important mechanism of cuproptosis resistance in tumors. In colorectal cancer and lung cancer models, fibroblast growth factor receptor 4 (FGFR4) has been shown (Zhong et al., 2026) to inhibit the activity of the deubiquitinase CYLD, resulting in ubiquitin–proteasome-dependent degradation of FDX1 and diminished sensitivity of tumor cells to cuproptosis. Concomitantly, FGFR4 degrades STING protein and suppresses type I interferon-mediated antitumor immunity, providing a new avenue for co-targeting receptor tyrosine kinases and cuproptosis. Whether this pathway participates in cuproptosis resistance and immune microenvironment remodeling in triple-negative breast cancer remains to be further investigated.

## 3 Multiple regulatory roles of FDX1 in cuproptosis

### 3.1 FDX1 as the key initiator of cuproptosis

FDX1 catalyzes the critical rate-limiting step in cuproptosis initiation by reducing Cu^2+^ to Cu^+^. Compared with Cu^2+^, Cu^+^ exhibits stronger cytotoxicity, as it can rapidly penetrate the mitochondrial membrane and accumulate extensively within mitochondria, thereby leading to lipoylated protein damage and destabilization of iron-sulfur cluster proteins; this represents the core toxic form of copper ions (Hsiao et al., 2025). Related studies have confirmed (Tsvetkov et al., 2019; Tang et al., 2022), that copper ionophores (such as elesclomol) can promote copper ion influx, while FDX1 enables the recycling of these ionophores by reducing Cu^2+^ bound to elesclomol, thereby further amplifying the cytotoxicity of copper ions and accelerating the cuproptosis process (Figure 1 (Sun et al., 2025; Li SR. et al., 2022)).

**FIGURE 1:**
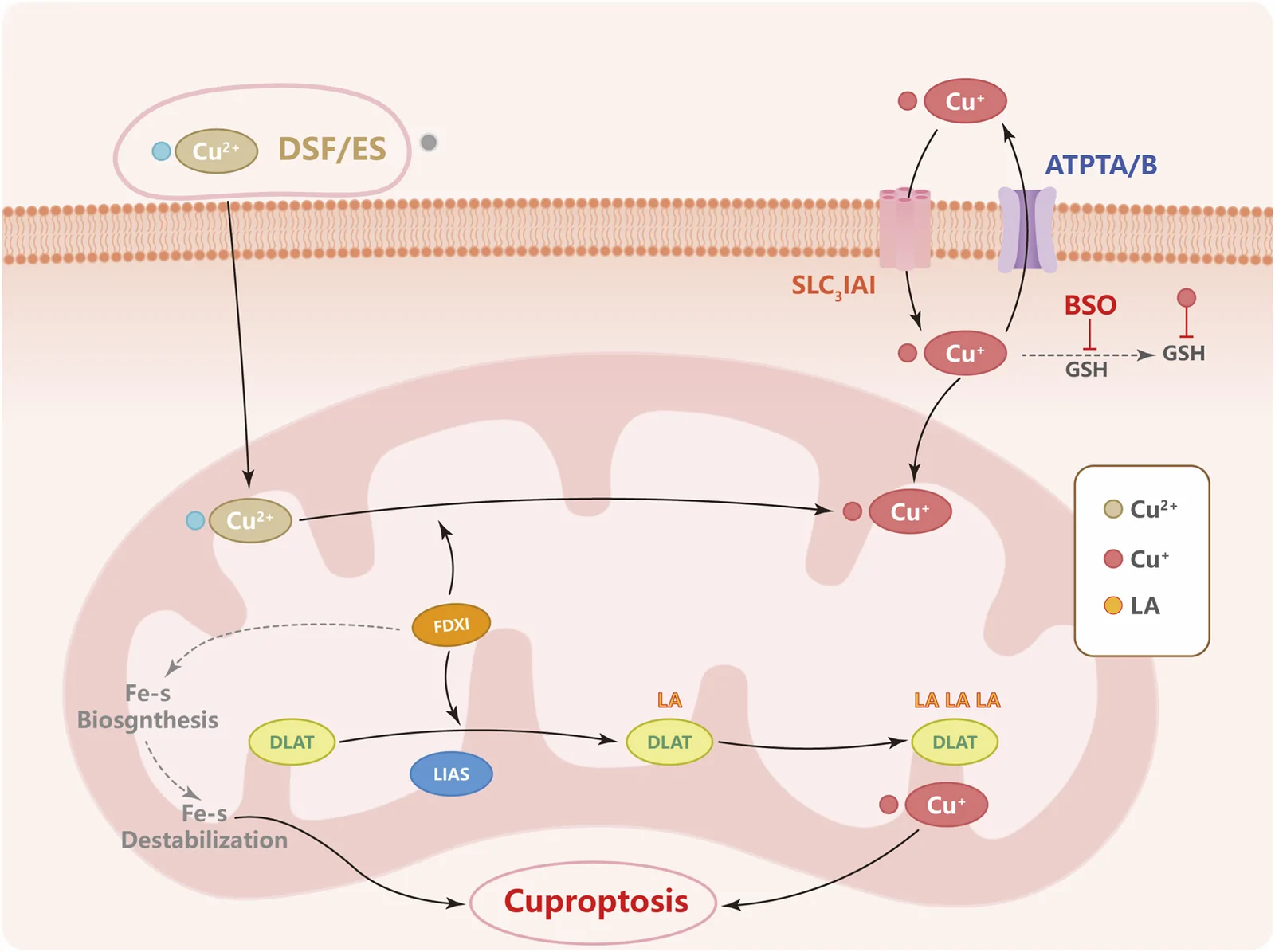
Mechanism of Cuproptosis. Extracellular Cu^2+^ enters cells through the SLC31A1 transporter, while ATP7A/B mediates copper efflux. Intracellular Cu^2+^ is reduced to Cu^+^ by FDX1 (ferredoxin 1). GSH buffers intracellular copper, and BSO inhibits GSH synthesis, promoting copper accumulation. Cu^+^ directly binds and oligomerizes lipoylated DLAT, causing lipoylated protein aggregation (LA). Meanwhile, copper accumulation destabilizes Fe-S cluster proteins *via* FDX1 involvement in Fe-S biosynthesis. Together, DLAT aggregation and Fe-S protein destabilization trigger cuproptosis. Adapted Li et al. (2022).

### 3.2 Regulatory role of FDX1 in lipoylation modification

Abnormal lipoylation modification and subsequent oligomerization represent one of the core hallmarks of cuproptosis, and FDX1 serves as the critical upstream regulator of lipoylation. FDX1 promotes the functional interaction between lipoic acid synthase (LIAS) and the lipoyl carrier protein GCSH, and provides the essential electrons required for LIAS-catalyzed lipoylation reactions, thereby driving the lipoylation modification of key enzymes in the tricarboxylic acid (TCA) cycle. Importantly, the FDX1-mediated functional interaction between LIAS and GCSH depends mainly on electron donation by FDX1 and on the maintenance of cellular iron–sulfur cluster homeostasis; this interaction *per se* does not require copper ions. Copper acts downstream of lipoylation by binding to already lipoylated DLAT and DLST, thereby inducing their oligomerization and triggering cuproptosis (Dreishpoon et al., 2023). Among these, dihydrolipoyl transacetylase (DLAT) and dihydrolipoyl succinyltransferase (DLST) are the most critical—the lipoylation modifications of these two enzymes constitute the direct binding targets for copper ions, and their modification levels directly determine cellular sensitivity to cuproptosis (Tsvetkov et al., 2022; Li SR. et al., 2022). Studies have confirmed (Li SR. et al., 2022), that loss of FDX1 function leads to impaired activity of lipoylation-dependent enzymes such as DLAT and DLST, with a significant decrease in lipoylated protein levels. Copper ions, deprived of their binding targets, fail to induce cell death, ultimately resulting in cellular resistance to cuproptosis; this is also accompanied by metabolic abnormalities including loss of cellular respiratory function and increased sensitivity to glucose starvation. Furthermore, FDX1 can also participate in the cytotoxic effects of copper ionophores through the regulation of lipoylation modification (Peng et al., 2026), Copper ionophores such as elesclomol directly target lipoylation modifications, and the FDX1-mediated lipoylation process can enhance the anti-tumor efficacy of such ionophores.

### 3.3 Association of FDX1 with iron-sulfur cluster protein homeostasis

Disruption of iron-sulfur (Fe-S) cluster protein homeostasis is one of the core mechanisms of cuproptosis. FDX1 primarily participates in cuproptosis regulation by reducing Cu^2+^ to Cu^+^, whereas Fe-S cluster biogenesis under physiological conditions is predominantly mediated by FDX2 (with FDX1 playing an auxiliary role). Physiologically, when FDX1 is absent, FDX2 can provide electrons to the ISC complex and thereby achieve partial compensation at the level of Fe–S cluster synthesis. However, FDX2 is unable to replace FDX1 in driving LIAS-dependent lipoylation. This compensation is restricted to Fe–S cluster metabolism and fails to rescue lipoylation deficiency (Dreishpoon et al., 2023). During cuproptosis, Cu^+^ generated by FDX1 binds to lipoylated proteins (such as DLAT), inducing their abnormal oligomerization, which subsequently leads to mitochondrial proteotoxic stress and respiratory dysfunction. This process indirectly disrupts the biogenesis and stability of Fe-S cluster proteins, triggering mitochondrial membrane potential collapse and reactive oxygen species (ROS) burst. When excessive ROS accumulates, oxidative stress is induced, and oxidative stress is mechanistically linked to the cuproptosis pathway. In an animal study (Nasirmahalleh et al., 2025), high-fat diet-induced obese mice exhibited copper accumulation in the liver and kidneys. Tissue copper accumulation upregulated FDX1 expression, accelerated DLAT lipoylation, and drove cuproptosis. Oxidative stress further amplified the transcriptional activation of FDX1/DLAT, thereby aggravating mitochondrial damage and cell death. Therefore, in the obese state, copper overload and oxidative stress may constitute a vicious cycle。Additionally, FDX1 itself possesses the capability to support Fe-S cluster synthesis (albeit with lower efficiency than FDX2), and its functional loss simultaneously impairs both lipoylation modification and Fe-S cluster homeostasis, creating a synergistic lethal effect (Lu et al., 2025; Sheftel et al., 2010).

### 3.4 FDX1 as a critical link between cuproptosis and antitumor immunity

(Lei et al., 2026) established a bidirectional positive feedback loop between cuproptosis and antitumor immunity: cuproptosis acts as an immunogenic cell death modality that releases DAMPs to activate CD8^+^ T cells, while IFN-γ secreted by CD8^+^ T cells directly binds the FDX1 promoter through STAT1–IRF1, upregulating FDX1 transcription and increasing tumor cell sensitivity to cuproptosis. Cuproptosis inducers combined with PD-L1 blockade can overcome primary and acquired immunotherapy resistance, and FDX1 shows potential as a predictive biomarker for immunotherapy. (Gao and Xing, 2026) subsequently published a commentary on these findings, further summarizing their translational value and the unresolved scientific questions. This mechanism provides a theoretical rationale for combining cuproptosis with immunotherapy; nevertheless, whether this loop contributes to immune microenvironment remodeling in TNBC and whether it can be harnessed to improve TNBC responses to immunotherapy remain to be directly validated in TNBC systems. In a colorectal cancer model (Wu et al., 2026), high FDX1 expression in tumor cells was found to exert immunosuppressive effects *via* the FDX1–DICER1–DMBT1 axis; FDX1 knockdown relieved DMBT1 mRNA degradation, promoted DMBT1 secretion, mitigated CD8^+^ T cell exhaustion, and enhanced antitumor immunity, indicating that FDX1 exerts complex bidirectional biological effects. However, this conclusion has not yet been validated in TNBC.

## 4 Research progress on the correlation between FDX1 and triple-negative breast cancer

### 4.1 Expression characteristics of FDX1 in triple-negative breast cancer

As the core regulatory factor of the cuproptosis pathway, FDX1 exhibits remarkable complexity and heterogeneity in its expression in triple-negative breast cancer (TNBC). Compared with normal breast tissue, both mRNA and protein levels of FDX1 are generally downregulated in TNBC tumor tissues, and this low expression is closely associated with poor patient prognosis (Sun et al., 2025). However, single-cell sequencing analyses have revealed (Sun et al., 2026), the existence of an FDX1-high epithelial cell subpopulation (FDX1^+^) within the TNBC tumor microenvironment, whose FDX1 levels are significantly higher than those of corresponding cell populations in other breast cancer subtypes. In TNBC, FDX1 displays an apparently paradoxical pattern—overall downregulation at the tissue level yet focal high expression in a specific epithelial subpopulation. This discrepancy is fundamentally attributable to tumor spatial and cellular heterogeneity, and is further shaped by inherent differences between bulk RNA-seq and scRNA-seq. Bulk RNA-seq captures the averaged superposition of signals from all cells within a whole tumor tissue block; when FDX1 is downregulated in most tumor cells, the bulk profile reflects overall FDX1 downregulation. scRNA-seq, in contrast, resolves transcriptional information at single-cell resolution, allowing a minor epithelial tumor cell subpopulation to be separated from mixed tumor tissue and this small FDX1^+^ population to be identified. Because this FDX1^+^ epithelial subpopulation constitutes only a fraction of tumor cells, its high-expression signal is obscured in bulk sequencing by the signals from the numerous FDX1-low cells. Thus, the two findings are not in conflict.

### 4.2 Molecular mechanisms by which FDX1 regulates malignant progression of triple-negative breast cancer

AKT1-FDX1 Phosphorylation Axis-Mediated Cuproptosis Resistance: Although copper ion levels are significantly higher in TNBC tissues than in normal tissues, tumor cells exhibit substantial resistance to cuproptosis. Recent studies have demonstrated that this resistance originates from the functional regulation of FDX1 by the AKT1 signaling pathway (Sun et al., 2025); Upon activation by high levels of copper ions, AKT1 kinase directly binds to FDX1 and phosphorylates it at serine 63 (Ser63). This residue resides within the structural domain of FDX1 responsible for electron transfer and protein–protein interactions. Phosphorylation adds a negatively charged phosphate group that alters the local conformation and electrostatic properties of FDX1, impairs its electron transfer function, and diminishes its capacity to mediate lipoylation of target proteins such as DLAT, consequently interrupting the cuproptosis pathway. Site-directed mutagenesis demonstrated that substituting Ser63 with alanine (S63A) prevents phosphorylation at this site, reverses AKT1-induced cuproptosis resistance, and restores cuproptosis sensitivity in TNBC cells (Sun et al., 2025). While FDX1 is inherently a key factor in inducing cuproptosis, this AKT1-mediated phosphorylation abrogates its cuproptosis-inducing capacity, rendering TNBC cells resistant to cuproptosis and leading to therapeutic drug resistance. This discovery provides an important theoretical basis for the development of combination therapeutic strategies employing “AKT1 inhibitors plus copper ionophores,” opening new avenues for overcoming treatment resistance in TNBC.

FDX1-Mediated Metabolic Reprogramming: AKT1-mediated FDX1 phosphorylation not only blocks cuproptosis but also profoundly reshapes the energy metabolism pattern of TNBC. Phosphorylated FDX1 significantly suppresses mitochondrial oxidative phosphorylation while driving tumor cells toward glycolytic metabolism (Sun et al., 2025), Glycolysis serves as a rapid pathway for tumor cells to acquire energy and biosynthetic precursors (i.e., the classic Warburg effect), which directly supports the rapid proliferation, migration, and invasion of cancer cells. Notably, the specific claim that AKT1–FDX1 phosphorylation directly drives glycolytic reprogramming currently rests on a single report and has yet to be independently reproduced. Nevertheless, extensive related evidence has shown that functional inactivation of FDX1 impairs mitochondrial lipoylation, suppresses oxidative phosphorylation, and elicits compensatory upregulation of glycolysis in tumor cells—a phenotype consistent with the Warburg effect—thus providing indirect support for this regulatory axis.

FDX1-Mediated Immune Microenvironment Remodeling and Evasion: Single-cell RNA sequencing analyses have revealed (Sun et al., 2026), that FDX1^+^ epithelial cells are significantly enriched in TNBC tissues, and this cell population is closely associated with reduced CD8^+^ T cell infiltration, suggesting that FDX1 participates in constructing an immunosuppressive tumor microenvironment. Clinical sample analyses have further confirmed (Dongdong et al., 2025), that FDX1 expression levels are significantly correlated with the immune cell infiltration characteristics within the tumor microenvironment, collectively forming an “immune-privileged” environment conducive to tumor growth. Functional experiments have demonstrated (Sun et al., 2026), that high FDX1 expression not only directly enhances the proliferation, colony formation, migration, and invasion capabilities of TNBC cells, but also significantly suppresses the activation, proliferation, and cytotoxic functions of CD8^+^ T cells through intercellular interactions. This implies that even when immune cells infiltrate the tumor periphery, their anti-tumor functions are effectively suppressed by FDX1. In the context of TNBC, however, evidence that FDX1 regulates these molecules and directly inhibits CD8^+^ T cells is still limited, making this an important unmet research need in the field.

## 5 Clinical translational value of FDX1 in triple-negative breast cancer

### 5.1 Value as a diagnostic biomarker

As the core regulatory molecule of cuproptosis, FDX1 expression levels can indirectly reflect the copper metabolic activity of tumor tissues. TNBC is characterized by high copper ion levels, and FDX1 detection can provide a molecular basis for evaluating tumor copper load status, thereby guiding the screening of appropriate populations for cuproptosis induction therapy. Single-cell RNA sequencing analyses have shown (Sun et al., 2026), that compared with other breast cancer subtypes (Luminal A, Luminal B, and HER2-positive), TNBC tissues exhibit significant enrichment of FDX1^+^ epithelial cells, with FDX1 expression levels significantly higher than those in adjacent normal tissues. This distinctive expression profile renders FDX1 a potential auxiliary diagnostic biomarker for TNBC; particularly in cases where needle biopsy specimens are limited, immunohistochemical detection of FDX1 expression can assist in confirming the diagnosis of TNBC. To date, this conclusion rests solely on retrospective bioinformatics analyses and small-sample clinical validation. Large-scale, multicenter, prospective clinical evidence is still lacking, and both the diagnostic performance and clinical utility of this marker remain to be further validated.

### 5.2 Therapeutic potential of targeting FDX1 in TNBC

FDX1 exerts dual oncogenic effects in TNBC: on one hand, AKT1 phosphorylation abrogates its cuproptosis-inducing capacity, enabling tumor cells to evade death in a high-copper environment; on the other hand, phosphorylated FDX1 is repurposed to promote aerobic glycolysis, fueling tumor proliferation. Given the multi-dimensional translational value of FDX1 in TNBC, targeted therapeutic strategies should focus on three levels: ① directly blocking the oncogenic functions of FDX1; ② inhibiting its upstream kinase AKT1; and ③ reversing downstream cuproptosis resistance and glycolytic activation (Figure 2 (Sun et al., 2025; Li SR. et al., 2022)).

**FIGURE 2:**
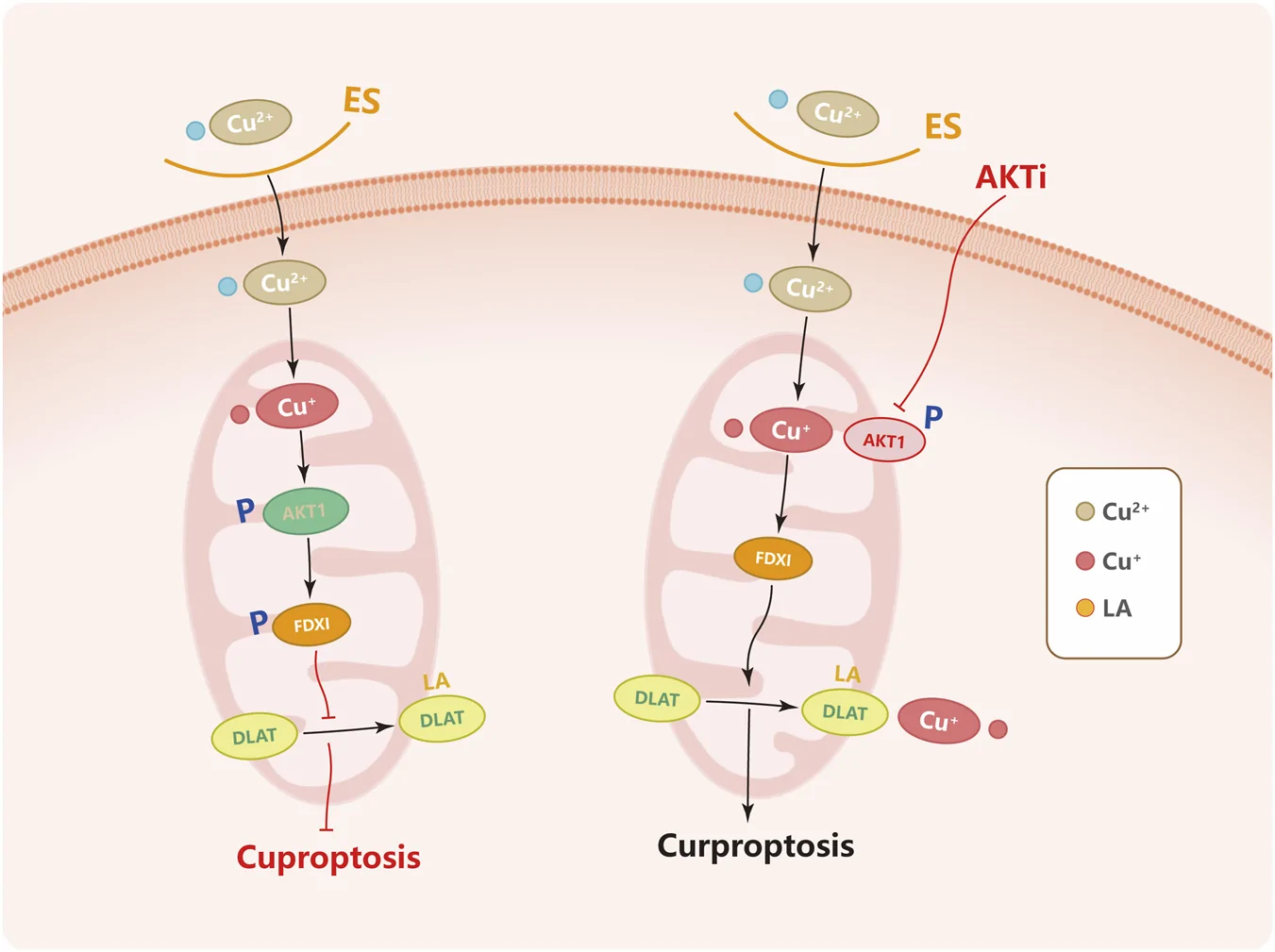
Schematic diagram of the phosphorylation mechanism by which the AKT1 signaling pathway regulates cuproptosis.Left panel: ES (Elesclomol) acts as a copper ionophore, promoting extracellular Cu^2+^ entry into the cell; intracellular Cu^2+^ is reduced to Cu^+^; Cu^+^ overload activates AKT1 (phosphorylated, marked as P); phosphorylated AKT1 further phosphorylates FDX1; phosphorylated FDX1 subsequently inhibits DLAT lipoylation, ultimately conferring resistance to cuproptosis.Right panel: ES (Elesclomol) acts as a copper ionophore, promoting extracellular Cu^2+^ entry into the cell; intracellular Cu^2+^ is reduced to Cu^+^; AKTI (AKT inhibitor) suppresses AKT1 phosphorylation; unphosphorylated AKT1 further activates FDX1; activated FDX1 promotes DLAT lipoylation (LA), and lipoylated DLAT functions normally, ultimately leading to cuproptosis. Adapted Sun et al. (2025).

Combination of AKT1 Inhibitors and Copper Ionophores: AKT1 inhibitors restore the cuproptosis-inducing function of FDX1 by blocking its phosphorylation; copper ionophores, in turn, elevate intracellular copper levels to provide the necessary conditions for cuproptosis. The efficacy of this combination strategy has been preliminarily validated in preclinical studies: the AKT1 inhibitor MK-2206 combined with the copper ionophore Elesclomol synergistically restored the sensitivity of TNBC to cuproptosis in both *in vitro* and *in vivo* experiments (Sun et al., 2025). AKT inhibitors have evolved from first-generation pan-inhibitors (such as Capivasertib and Ipatasertib) to highly selective ATP-competitive inhibitors (such as MK-2206 and TAS-117), with their therapeutic value in breast cancer, particularly TNBC, becoming increasingly prominent. Capivasertib is the first approved AKT inhibitor, demonstrating significant efficacy in the treatment of hormone receptor-positive advanced breast cancer (Turner et al., 2023),thereby providing an important reference for TNBC therapy. Studies have confirmed (Chica-Parrado et al., 2024) hat the combination of CDK4/6 inhibitors and AKT inhibitors exhibits significant synergistic anti-tumor activity in the LAR subtype of TNBC: this regimen simultaneously blocks cell cycle progression and PI3K/AKT survival signaling, overcoming the resistance induced by CDK4/6 monotherapy through AKT feedback activation, thus providing crucial preclinical evidence for this specific TNBC subtype; however, its clinical efficacy and safety still require validation through prospective clinical trials. CAPItello-290 (Schmid et al., 2026) is a Phase III clinical trial evaluating the efficacy of Capivasertib in TNBC, with results currently pending. At present, the synergistic anti-TNBC effect of AKT1 inhibitors combined with copper ionophores has been demonstrated only in preclinical studies at the cellular and animal levels. No human clinical trial has yet confirmed the efficacy or safety of this combination, leaving a clear translational gap between basic research and clinical application. Multiple unresolved challenges must be addressed before this novel combination therapy can reach the clinic. Regarding drug toxicity, for instance, the combination of copper ionophores with AKT inhibitors may exacerbate toxicity to normal tissues, leading to systemic adverse effects such as hepatorenal injury and hematopoietic abnormalities. From a pharmacokinetic perspective, the two drug classes differ in their *in vivo* absorption, distribution, metabolism, and excretion profiles, and co-administration may result in drug–drug interactions that compromise overall therapeutic efficacy and elevate medication risk.Future efforts should further explore combination strategies of AKT1 inhibitors and copper ionophores, with the aim of breaking through the therapeutic bottleneck in TNBC.

Nanodelivery Therapeutics: (Guo et al., 2023) developed a reactive oxygen species (ROS)-responsive nanoparticle (NP@ESCu), achieving for the first time the organic integration of cuproptosis induction with anti-PD-L1 immunotherapy. This system synergistically induces cuproptosis through the combination of the copper ionophore elesclomol and Cu^2+^, and upon combination with anti-PD-L1 antibody, successfully transforms the immunosuppressive tumor microenvironment into an immunogenic microenvironment, significantly enhancing anti-tumor efficacy. This approach holds important reference value for the treatment of “cold tumors” such as triple-negative breast cancer. (Li et al., 2024) designed a core-shell structured nanoparticle CuP/Er, which achieves for the first time the synergistic activation of cuproptosis and ferroptosis through pH-responsive release of copper ions and the ferroptosis inducer erastin (Er). Its mechanism lies in enhancing tumor cell sensitivity to copper ions through an “anti-Warburg effect,” thereby amplifying the cuproptosis effect. In a TNBC (4T1) model, this nanoplatform not only directly kills tumor cells but also activates anti-tumor immune responses and upregulates PD-L1 expression, significantly enhancing the efficacy of anti-PD-L1 immune checkpoint blockade therapy and effectively inhibiting tumor growth and metastasis. As noted in related nanomedicine reviews (Lu et al., 2026), FDX1 and mitochondrial oxidative phosphorylation status serve as core markers determining tumor sensitivity to cuproptosis-targeting nanoformulations and can inform the design of iron–copper co-activating nanomedicines, offering new therapeutic avenues for TNBC. Nevertheless, nanodelivery therapies to date have been validated only in animal models, and critical clinical translation issues—such as drug targeting efficiency, *in vivo* safety, and human pharmacokinetic characteristics—remain unresolved. At this stage, they can serve only as a foundation for basic research into novel TNBC treatment strategies. Substantial basic optimization and early-phase clinical investigation will be required to progressively establish their clinical translational feasibility.

## 6 Conclusion and perspectives

As the core regulatory factor of cuproptosis, FDX1 plays pivotal roles in metabolic reprogramming, immune microenvironment remodeling, and therapeutic resistance in TNBC; however, current research still faces several critical bottlenecks that urgently require breakthroughs. Existing studies predominantly rely on retrospective analyses of public databases such as TCGA and GEO, along with *in vitro* experiments, lacking validation from prospective clinical cohorts; consequently, the clinical value of FDX1 as a diagnostic biomarker or therapeutic target has yet to be established. Second, FDX1 is highly expressed in the liver and adrenal glands, representing a safety concern and a source of off-target effects that must not be overlooked in targeted intervention. Physiologically, FDX1 localizes to mitochondria, where it regulates key processes including iron–sulfur cluster synthesis, mitochondrial respiration, and substrate metabolism, thereby sustaining normal physiological functions such as hepatic metabolic homeostasis and adrenal steroid hormone synthesis.The mitochondrial respiratory function of FDX1 overlaps between tumor cells and normal tissues (such as liver and adrenal gland), and no tissue-specific isoforms or tumor-specific conformations have been reported to date; traditional small-molecule inhibitors are unable to distinguish pathological from physiological states of FDX1, posing risks of systemic toxicity upon systemic administration. Future studies will need to prioritize breakthroughs in tissue-specific targeted delivery and precision tumor-targeted regulation, and to develop tumor-selective FDX1 modulators that circumvent off-target effects in normal tissues with high FDX1 expression, such as the liver and adrenal glands, so as to minimize systemic toxicity.The expression heterogeneity and regulatory networks of FDX1 across different TNBC molecular subtypes remain incompletely elucidated. Furthermore, specific small-molecule drugs targeting FDX1 are still in early developmental stages, while copper ionophores face pharmaceutical challenges including poor *in vivo* stability and off-target toxicity.

With the advancement of precision medicine technologies, future directions should include: establishing standardized FDX1 immunohistochemical detection and serum antibody detection systems, and validating its prognostic predictive value in multi-center prospective cohorts; simultaneously, the development of novel FDX1-targeted modulators, precision nanodelivery systems, and individualized combination therapeutic regimens. As precision medicine technologies continue to progress, FDX1 is expected to transition from basic research to clinical application, providing novel targets and strategies for the precision diagnosis and treatment of TNBC.
